# Assessment of Whole Body and Local Muscle Fatigue Using Electromyography and a Perceived Exertion Scale for Squat Lifting

**DOI:** 10.3390/ijerph15040784

**Published:** 2018-04-18

**Authors:** Imran Ahmad, Jung-Yong Kim

**Affiliations:** Department of Industrial Management Engineering, Hanyang University, Ansan 15588, Korea; imran86@hanyang.ac.kr

**Keywords:** electromyography, principal component analysis, whole body fatigue, squats, Borg scale, musculoskeletal disorders, assessment, muscle

## Abstract

This research study aims at addressing the paradigm of whole body fatigue and local muscle fatigue detection for squat lifting. For this purpose, a comparison was made between perceived exertion with the heart rate and normalized mean power frequency (NMPF) of eight major muscles. The sample consisted of 25 healthy males (age: 30 ± 2.2 years). Borg’s CR-10 scale was used for perceived exertion for two segments of the body (lower and upper) and the whole body. The lower extremity of the body was observed to be dominant compared to the upper and whole body in perceived response. First mode of principal component analysis (PCA) was obtained through the covariance matrix for the eight muscles for 25 subjects for NMPF of eight muscles. The diagonal entries in the covariance matrix were observed for each muscle. The muscle with the highest absolute magnitude was observed across all the 25 subjects. The medial deltoid and the rectus femoris muscles were observed to have the highest frequency for each PCA across 25 subjects. The rectus femoris, having the highest counts in all subjects, validated that the lower extremity dominates the sense of whole body fatigue during squat lifting. The findings revealed that it is significant to take into account the relation between perceived and measured effort that can help prevent musculoskeletal disorders in repetitive occupational tasks.

## 1. Introduction

In the modern industrial working environment, workers are less susceptible to heavy lifting tasks as compared to the old industrial manual lifting techniques; however, they are more exposed to physically demanding repetitive tasks [1,2]. The muscle fatigue developed as a result of such repetitive tasks has been addressed by many researchers using physiological [3,4,5,6] and psychological [7,8] approaches. Such fatigue has been linked to perceived exertion, which a worker perceives as whole body fatigue. Thus, it is important to maintain a balance between work load and physical capacity [9]. Among such physically demanding tasks, squatting is one of the tasks encountered by industrial workers that exposes the workers to a high risk of developing musculoskeletal disorders [10]. Therefore, it is imperative that work schedules for manual material handling tasks be designed in such a way that cumulative fatigue developed as result of repetition may be avoided. This is only possible if the assessment of exertion levels can be measured on site [1]. Cumulative and long term fatigue is governed by central and peripheral factors. This makes it important to evaluate the underlying central and peripheral factors that cause an elevated level of perceived whole body fatigue. The sense of effort in the form of muscular pain or loss of muscle force in the form of fatigue is important to monitor as these are one of the causes of musculoskeletal disorders (WMSD) [11,12]. Fatigue monitoring systems have been made possible through wearable sensors [13]; however, their relation with perceived effort still needs to be addressed.

Whole body fatigue is a complicated phenomenon as the factors that contribute to its estimation need to be investigated. There has been an increasing trend in using perceived fatigue to guide exercise testing and also to find onsite exertion levels in individuals with spinal cord injuries and factory workers using manual lifting techniques [11]. Borg [14,15] attempted to address the issue of whole body fatigue by developing a scale, ranging from 0 to 10 (Borg CR-10). One of the difficulties that is encountered while assessing whole body fatigue is inter-individual comparison. Different methodologies that have been used as an index of whole body fatigue are the heart rate and oxygen exchange rate [11]. However, it is still not clear whether muscular level fatigue using electromyography (EMG) for perceived whole body fatigue has any association with a particular muscle type or group of muscles. Thus, there is a need to adopt a different measurement methodology where the whole body fatigue is confused with local muscle fatigue.

Accurate measurements are important to form a basis for reliable assessment. EMG has long been used to assess the fatigue level during manual lifting [16]. It has proven to be a valid tool for evaluating muscle fatigue incorporating different task variables [3,16,17,18]. Different models have been proposed by using different indexes of EMG as an input variable [19,20] that can predict muscle fatigue in terms of force. Among the noninvasive methods, surface electromyography (sEMG) has been widely used to quantify muscle fatigue [21,22]. Different indices of EMG have been used to assess the level of muscle activity and fatigability [23]. Among such parameters, the frequency domain parameters, mean, and median frequency have been used by many researchers to assess the level of local fatigue [24]. However, one drawback of the median and mean frequency is that these parameters have proven to be quite insensitive at low loads [25]. Igor et al. [23], in a cycle ergometer test starting with a 12.5 W/min initial load from zero to 500 dynamic muscle contractions, showed that the slope versus the standard distance of principal component analysis (PCA) presented the differences in the sensitivity levels of both methods. Therefore, an alternate method is required to address the issues of muscle fatigue identification through EMG. Among such methods, PCA is commonly used to reveal the features in neuromuscular patterns [26].

PCA is often employed in multivariate clinical settings and uses linear transformation applied to raw data that are represented as the principal components. These components are in the direction of maximum variance and are orthogonal to each other. This method helps in representing the data in N-dimensional space to P-dimensions using the principal components where P < N [27]. This helps in reducing the dimensionality of the data while retaining most of the variations in the data set [28]. Dhindsa et al. used PCA for the selection of significant muscles for force estimation using sEMG where a significant number of muscles that showed the maximum variances were selected. Here, 92.2% variance was explained by using just two principal components [29]. Similarly PCA has been used in the classification of patterns in gait analysis, as well as between control groups and patients with lower limb fractures using ground reaction forces [30].

The assessment of local muscle fatigue and whole body fatigue has been addressed by many studies; however, the assessment has been performed separately for the two phenomena. The relation between these two phenomena (i.e., the local muscle fatigue and the whole body fatigue during a lifting task) still needs to be identified. Another crucial factor to be evaluated is the domination of these two phenomena over time as the body undergoes fatigue. In order to evaluate the level of fatigue and the relation between whole body and local muscle fatigue, the methodology employed plays a crucial role. Secondly, an alternate method for evaluating such fatigue needs to be validated as there have been studies reporting the sensitivity issues in evaluations of fatigue at low levels.

Therefore, this study aims to propose a new way of observing whole body fatigue and its relation to local muscle fatigue based on a perceived exertion scale and the application of PCA. Two different weights were used for squat lifting to further analyze the effects of weights on perceived exertions. For the perceived exertion readings, the body was segmented into three regions: lower, upper, and whole body fatigue. Heart rate and sEMG signals were recorded for local and whole body fatigue analysis.

## 2. Materials and Methods

### 2.1. Subjects

Twenty five male subjects participated in this study (mean age 30 ± 2.2 years, weight 73 ± 9.94 kg, stature 177 SD ± 6.37 cm and BMI 23.98 ± 2.63). Each subject signed a document of informed consent written according to Korean good clinical practices and Bioethics and safety act approved by the Hanyang University, Institutional Review Board (IRB) committee, South Korea. The exclusion criteria for this study included subjects with no history of low back pain, any joint-related disease, and visual impairment including colorblindness. The subjects volunteered from the student population of the university.

### 2.2. Muscle Selection and Location of Electrodes

Bipolar EMG (Ag-Ag/Cl) electrode configuration was used with an inter electrode distance of 20 mm. Before the placement of the electrode, the skin was shaved and cleaned with alcohol. The skin was left to dry for 60 s to reduce the myoelelctrical impedance. Surface EMG signals recorded from eight muscles included the: (1) bicep femoris (BF); (2) vastus lateralis (VL); (3) anterior deltoid (AD), (4) rectus femoris (RF); (5) middle deltoid (MD); (6) upper trapezius (UT); (7) gastrocnemius medialis (GS); and (8) supraspinatus (SP). To minimize the cross talk between the muscles, electrodes were placed carefully on the muscle belly. The protocols for the sensor location for this study followed the recommendations made by SENIAM [23,31]. For the bicep femoris, the electrode was placed at 50% on the line of the ischial tuberosity and the lateral epicondyle of the tibia. For the anterior deltoid, the electrodes were placed at one finger width away and anterior to the acromion. For the medial deltoid, the electrodes were placed 3 cm below the acromion, over the muscle bulk, aligned with the muscle fibers [32]. For the vastus lateralis, electrodes were placed at 2/3 on the line from the anterior spina iliaca superior to the lateral side of the patella. For the rectus femoris, the electrodes were placed at 50% on the line from the anterior spina iliaca superior to the superior part of the patella. For the gastrocnemius medialis, the subjects laid supine. The knee was extended and the foot flexed. An electrode was placed on the most prominent bulge of the muscle. For the upper trapezius, the electrodes were placed at 50% on the line from the acromion to the spine on vertebra C7. The supraspinatus electrodes were placed over the suprascapular fossa [32]. Maximum voluntary contraction for each muscle was attainted for the normalization of the EMG signal for each muscle.

### 2.3. Apparatus

An eight channel EMG system (ME6000 Bittium Bio signals, Mega electronics, Kuopio Finland Ltd.; Model ME6000, Kuopio, Finland) was used to acquire EMG signals with a sampling rate of 1024 Hz. The Ag-/AgCl bipolar configuration for each muscle was used with an electrode size of 10 mm and an inter electrode distance of 20 mm [22,31]. A polar heart rate monitor was used for the measurement of the Heart Rate-based beat-to-beat interval (RR interval). The reliability of the Polar H Series monitor for the RR intervals was verified by comparing it with the ECG monitor. The correlation (*r* = 0.99) of ECG readings with the heart rate monitor has been checked by many studies for its clinical use [11,33].

### 2.4. Pre-Processing of EMG Signal

**Figure 1:**
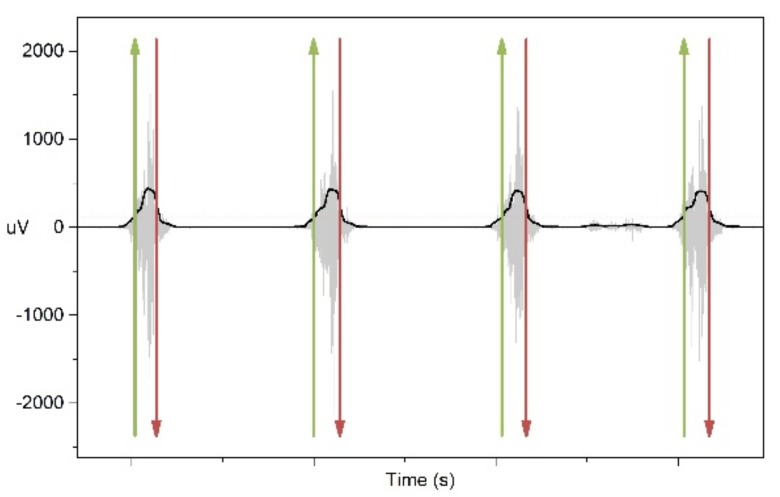
Muscle activation pattern of rectus femoris. The onset and offset of the muscle are designated by the arrows pointing upwards for the onset and downward for the offset.

The EMG signals were sampled at 1024 Hz and band pass filtered with cutoff frequencies of 10–500 Hz. The muscles were intermittently activated during the dynamic lifting. The activation pattern of each muscle was identified through a defined threshold level of the EMG signal until the 24 activations were observed. The beginning of each activation pattern was defined through the Root Mean Square (RMS) value with the attained threshold value and ended when the RMS value became inferior to the threshold value for the consecutive data points. MATLAB routines and Origin Pro were used for the processing and statistical analysis. A 250 ms window width was used with a Hanning window type for conversion to the frequency domain using the Fast Fourier transform [34]. Figure 1 shows the activation of the rectus femoris muscle of a subject. The onset and offset of the muscle are designated by the arrows pointing upwards for the onset and downward for the offset.

### 2.5. Principal Component Analysis

For the PCA method, the normalized EMG for the eight muscles was taken as eight features (variables) for the data matrix. PCA reduces the dimensionality of the data while retaining most of the variation in the data set. The PCA de-correlates the multivariate data by projecting the data onto a new coordinate system. For the *k* principal variables, which in this case were the eight muscles, the *k* principal components were generated. The covariance matrix was obtained by using the following equation [27]:

(1) $$S_{jk}=\frac{1}{N-1}\overset{N}{\underset{i=1}{\sum }}\left(x_{ik}-{\bar{x}}_{j}\right)\left(x_{ik}-{\bar{x}}_{k}\right)$$

Here, *N* is the number of the mean power frequency components from each subject and ${\bar{x}}_{j}$ and ${\bar{x}}_{k}$ are the mean values for the corresponding mean power frequencies *j* and *k*, respectively. After the mean-adjusted data, the eigenvalues and the eigenvectors of the covariance matrix were obtained. The eigenvalues were sorted in descending order. A scree-plot shows the explained variance in terms of the percentage. After the number of eigenvalues was evaluated, the transformation matrix was formed. The data was then projected onto the transformation matrix.

The principal components were calculated by the following equation:

(2) $$Sx_{p}=\lambda_{p}x_{p}$$

where, λ is the chosen eigenvalues and ***x*** is the corresponding eigenvector. The major muscles during the squatting for the two different types of weight lifting from the PCA analysis were designated as the muscles that had the highest absolute magnitudes ranging between zero and one. For this study, the first two modes of PCA were taken into account. The frequency of muscles that came in the first mode of PCA was counted as they had the highest eigenvalue.

## 3. Experiment Design

The experiment consisted of a lifting task parallel to the sagittal plane. The lifting weights of 4 kg and 8 kg were used as independent variables [3]. Subjects lifted a box (32 (width) × 40 (length) × 25 (height) cm, containing handles on either side), which was placed symmetrically in front of their feet, to an upright symmetrical standing position. These load levels were approximated to the average load levels observed in industry [35]. Normalized sEMG values from the eight muscles were taken as the dependent variables from which the slopes of the mean and frequency were used as estimators of fatigue for the muscles. The Borg scale for Peripheral Rates Perceived of Exertion (PRPE) and Central Rates of perceived Exertion (CRPE) was used to assess the level of perceived fatigue. A picture of the different regions of the body to be perceived by the subject and the table for the Borg CR 10 scale was displayed in front of the subjects during squats.

### 3.1. Procedure

**Figure 2:**
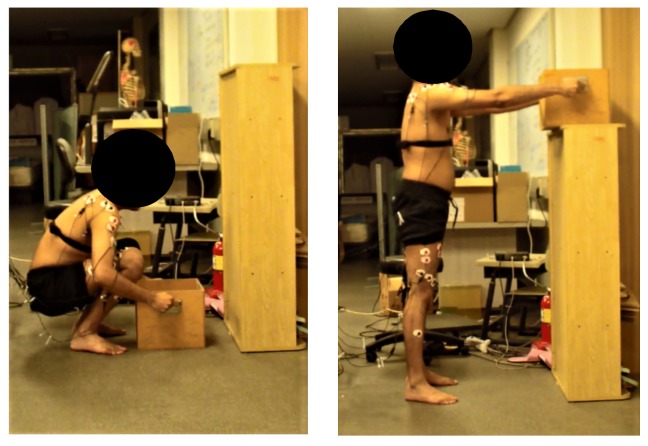
Symmetric lifting and lowering.

Participants abstained from eating three hours before the experiment. No trial was taken before 10 AM or 5 PM to reduce the effect of diurnal variations. The timings of the trial were fixed concerning the effect of time of the day on EMG parameters [36]. Each subject visited the lab twice for two sessions. During the first session, familiarization with the exercise, as well as the Borg Scale, was conducted. The subjects were asked questions during the pilot test to take into account the sensations of exertion in the legs and shoulder, and the overall exertion being felt in the whole body [37]. In the second session, the experiment was performed. For the experiment, it was ensured that the plantar surface of the foot was in full contact with the ground as shown in Figure 2. The subject’s posture for the start of the lift was adjusted using a goniometer so that each subject assumed the same posture at the start and end of the task. For the start position of the squat, the knee flexion was adjusted to be less than 45 degrees. The origin of the box was located at the center of gravity of the box. The frequency of the lift was one lift per ten seconds, with four seconds to complete lowering and lifting the weight to elbow height. A metronome was used to control the speed of the task. Each subject performed 24 sets of squats with a ten-second rest interval between each squat for 4 and 8 kg weights, respectively. The trials were randomized across the subjects. After every four squats, the subject’s peripheral and central exertion levels were assessed by asking the subject to point out his exertion level on a displayed Borg’s scale in front of the subject. At the end of the task, the subjects were asked to report their perceived exertion with their heart and lung area as central factors and separately for legs and arms as peripheral factors. EMG readings were recorded from the start of the task until the end of it.

### 3.2. Data Analysis

The mean and standard deviations were calculated for the upper, lower, and whole body ratings of the perceived exertions of the Borg scale. For the heart rate, the RR interval was monitored for each subject after four sets of squats. For 24 squats, a total of six readings were observed for the Borg scale and the heart rate. The filtered EMG signal was converted to the frequency domain and the mean power frequency was found using Fast Fourier Transform [38]. Window width was kept at 250 ms with 30% overlap. After obtaining the mean power frequencies for all sets of squats, the results were analyzed statistically.

### 3.3. Statistical Analysis

The ratings of perceived exertions were checked for internal consistency through the Cronbach alpha test. Test re-test reliability was checked through intra class correlation. Analysis of Variance (ANOVA) was used to compare the difference in perceiving upper, lower, and whole body fatigue between 4 and 8 kg squat lifting with a significance level of *p* < 0.05. For normalized mean power frequency (NMPF), the slope was found through a regression line by plotting against the number of squats. The slope revealed the level of fatigue of muscles as compared to the muscles of the same extremity. The slope for the heart rate was also compared to whole body fatigue. Pearson’s coefficient (*r*) was used to find the correlation of NMPF of each muscle with the Borg scale ratings for the upper and lower extremity of the body. The correlation between heart rate and the perceived whole body fatigue was also found. Two way mixed absolute agreement, intra-class correlation was also used as a reliability index for the Borg scale. After observing the key muscles through the slope method and finding the correlation, with respect to the two extremities and whole body, the results were further validated through PCA.

## 4. Results

The Cronbach alpha values for the 4 kg weight for the perceived exertions of the upper, lower, and whole body as indicated by the subjects were between 0.82 and 0.87, while for 8 kg, they were between 0.90 and 0.92. Higher internal consistency was observed for rating of perceived exertion for the 8 kg squat lifting weight because the majority of subjects reported identical exertion levels near the end of the task. The intra-class correlation ICC values for the Borg scale rating for 4 kg squat lifting were between 0.71 and 0.86, and for 8 kg, were 0.85–0.94 [39]. A paired *t*-test was used to check whether the mean of the perceived fatigue was significantly different for 4 and 8 kg squat lifting. It was observed that there was a significant difference (*p* < 0.05) between the two conditions of the weights (Table 1).

**Figure 3:**
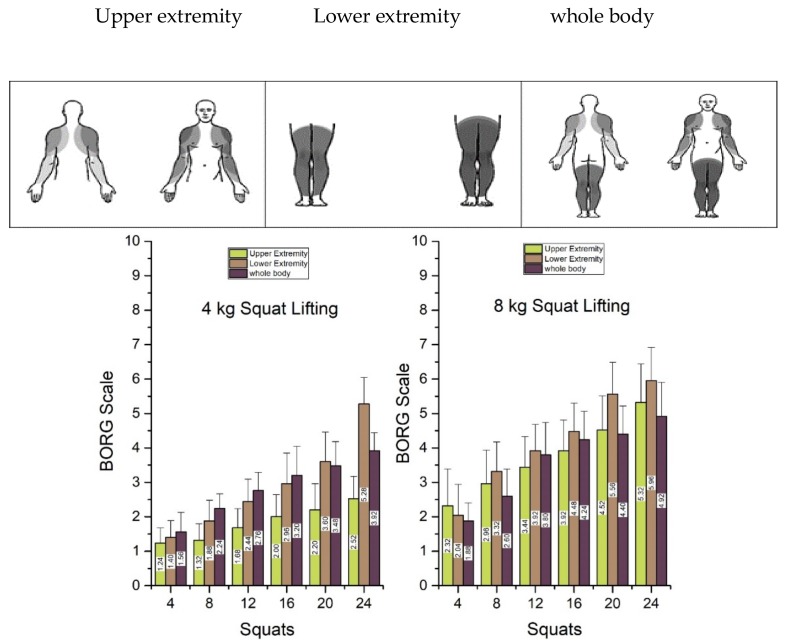
Borg scale readings for upper and lower extremity and whole body for the 4 kg and 8 kg weights. Grey areas are highlighted to represent the perceived regions for the response against the Borg scale.

Figure 3 shows that the RPE for 4 kg squat lifting surpassed that of the whole body and the upper perceived exertions until the 16th squat. Conversely, for the 8 kg weight, the RPE for the lower region surpassed both the upper and the whole body RPE after the eight squat. The ANOVA results for the 4 and 8 kg squat lifting for the three regions of the perceived exertions are shown in Table 1. It can be seen at the start and end sessions that the whole body fatigue between the two groups was not perceived differently by the subjects.

**Figure 4:**
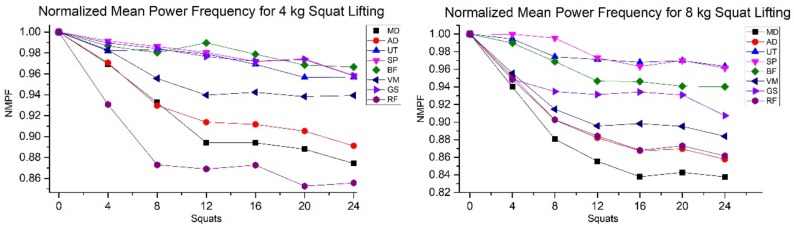
Normalized mean power frequencies (NMPF) for the eight muscles. Medial deltoid (MD), anterior deltoid (AD), upper trapezius (UT), supraspinatus (SP), bicep femoris (BF) vastus laterals (VS), gastrocnemius (GS), and rectus femoris (RF).

The regression line slopes of the heart rate and NMPF of the eight muscles with respect to squat sets for all eight were calculated (Table 1). The slope of the normalized heart rate was positive as it increases with respect to the exertion level, while for the NMPF, the slope decreased as there is a shift towards the left side in the power spectrum as a muscle experiences fatigue. The muscle with the maximum fatigue was detected through the highest negative slope of the regression line for the upper and lower extremity, respectively. Figure 4 shows the NMPF for the eight muscles for the 4 and 8 kg weight lifts. A decrease in the slope signifies the onset of muscle fatigue.

Table 2 shows that, for both types of weights, the medial deltoid and the rectus femoris muscle showed the maximum decrease in the NMPF for upper and lower extremities, respectively. As the steepness of slope is an indication of muscle fatigue [40], these two muscle were observed to experiencing greater fatigue compared to the others.

The NMPF was then correlated with ratings of perceived exertions (RPE) for the muscles of the upper and lower extremity and for the whole body, it was correlated with the heart rate (Table 3). The normalized heart rate and the RPE for the whole body showed the highest correlation (*r* = 0.96). For 4 kg, in the lower and the upper extremity, the supraspinatus (*r* = −0.96) and the vastus lateralis (*r* = −0.95) showed a significant relation. For 8 kg squat lifting, the Anterior deltoid (*r* = −0.88) and the Bicep femoris (−0.87) showed a significant relationship.

**Figure 5:**
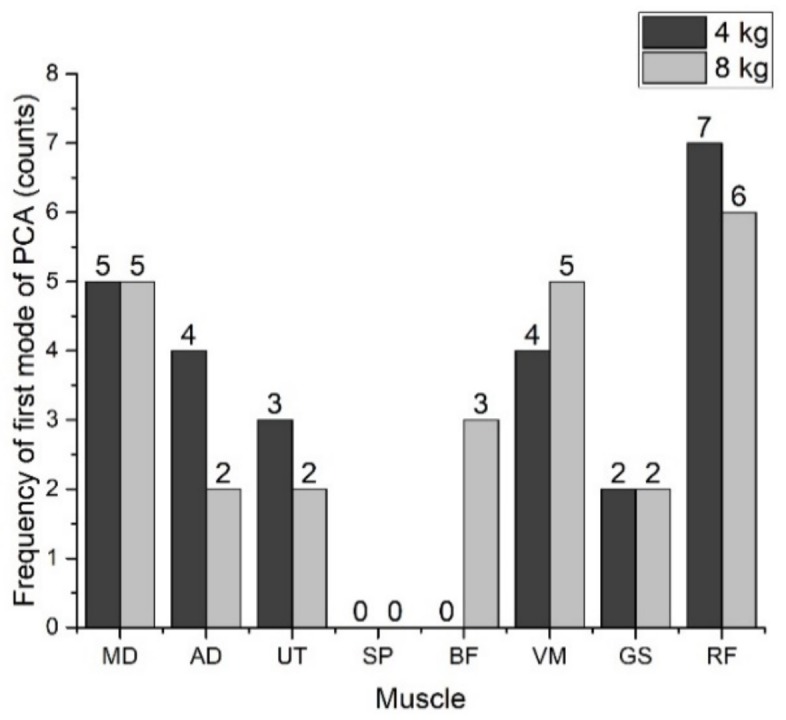
Principal muscles reviled by first mode of PCA for squat lifting for 4 and 8 kg weights. Medial deltoid (MD), anterior deltoid (AD), upper trapezius (UT), supraspinatus (SP), bicep femoris (BF) vastus laterals (VS), gastrocnemius (GS), and rectus femoris (RF).

Figure 5 shows the maximum frequency of the two muscles that appeared in the first mode of PCA, which were the medial deltoid and the rectus femoris muscles among all the 25 subjects. The rectus femoris muscle, having the highest frequency, revealed that as in the perceived exertion, the lower extremity was dominant over the whole body and the upper extremity. These results are in agreement with the results obtained from the perceived exertions scales. Although for the 8 kg squat lifting the slope of the NMPF of the medial deltoid was greater than that of the rectus femoris, the frequency of both the muscles is the same. Further results can be checked by taking into account the second and the third modes of PCA.

## 5. Discussion

The aim of this study was to assess body fatigue for repetitive industrial tasks by segmenting the body into regions of perceived exertions and finding the key muscles that are the main cause of fatigue in a particular region. For this purpose, the body was segmented into upper and lower regions of perceived exertion and whole body. The Borg scale and sEMG were used to assess the level of fatigue that accumulates and ultimately dominates any of the three regions of perceived exertion. The segmental approach of assessing whole body fatigue is a unique approach as previous studies have assessed fatigue levels by taking the body as a single unit or have taken just one region of the body, such as in a study by Hummel et al. [41], where only the upper trapezius was taken into account to find the relation between perceived fatigue and EMG. The correlation between perceived exertion and the EMG was *r* = 0.76., whereas in our research, the correlation for the upper trapezius was much higher (0.83). The segmental approach could be one of the reasons for this high correlation, as in the segmental approach the subjects were able to compare their regional fatigue with the whole body, which was lacking in most of the studies. The perceived region of exertion was further analyzed using sEMG. Previous studies have established the domination of peripheral factors in fatigue assessment [8,11], and a similar pattern was observed in this study. However, the combined approach of taking perceived exertion scales and sEMG readings on segmental body regions helped to generate a more accurate perceived and physiological assessment as compared to a single segment assessment.

### 5.1. Perceived Exertions

For the RPE, the Borg scale was used to evaluate which region of the body dominates for free dynamic squat lifting. The Table 4. Summarizes the evaluation of the sensation of domination for perceived exertion to be used as quantification of exertion based on extremity.

For the 24 sets of trials, for two different types of weights, it was observed that the lower extremity of the body dominated the perceived exertion as compared to the other two regions.

### 5.2. Mean Power Frequency and Heart Rate

To further analyze the task, EMG signals from eight muscles were obtained. The frequency domain data using the Fast Fourier transform were used to find the muscle that had the greatest decrease in the slope, thereby detecting the onset of greater fatigue as compared to the other muscles. Eight different muscles were chosen from the upper and lower extremity of the body that are activated during squat lifting. For both the 4 kg and 8 kg squat lifting, the medial deltoid and the rectus femoris muscles showed significant decreases in the slope as compared to the other six muscles, indicating the highest fatigue levels. The sEMG analysis was performed for the analysis of peripheral fatigue, while for the central determination of sensation, the heart rate was taken as the physiological variable. A study by Vahdat et al. used mean power frequency and found that multifidus and iliocostalis lumborum muscles were activated the most for 4 and 8 kg squats [3]. However, perceptual effort was still missing, which can yield different results, as shown in the current study. It was observed that for the medial deltoid muscle, the fatigue level was even greater; however, the fatigue perception as a sense of discomfort was indicating the lower extremity to be the dominating factor. This discrepancy between the two phenomena forms the basis of the fact that cognitive parameters should be considered when quantifying muscle fatigue.

### 5.3. Principal Component Analysis

The main advantage of PCA over the slope method for the detection of muscle fatigue was the extraction of the principal muscles. It was observed that, for the upper extremity, the medial deltoid had the maximum counts among all the subjects, while for the lower extremity, the rectus femoris exhibited the highest counts. Compared to both extremities, the rectus femoris displayed higher counts as compared to the medial deltoid. This is in agreement with the perceived exertion scale where the lower extremity was a dominating factor. A study by Charoenpanicha Net et al. used principal component analysis to find the principal muscles activated during the squat jump [42]. Other indices such as standard distance have also been used by many studies has and have the advantage of sensitivity [25]. However, again, these studies mostly employ the cycle ergometer as the assessment method. The current study has used principal component analysis for squat lifting which mimics the industrial tasks and thus a more realistic approach has been used.

The agreement of the results between the Borg scale readings, the slopes of the mean power frequency, and the PCA method signifies the fact that a combined approach of cognitive perception and sEMG can better detect the onset of cumulative fatigue. Secondly, the heart rate which was taken as the central factor of perceived exertion was not as dominant as the lower extremity of the body.

## 6. Conclusions

The results of this study showed that a combined approach of cognitive perception of fatigue, heart rate, and sEMG can better provide information for detecting fatigue compared to just individual parameters. This study can also help in designing a framework for fatigue detection through wearable sensors. This work is also important as it can help in developing a framework to design work load schedules for repetitive tasks.

## Figures and Tables

**Table 1:** ANOVA results of 4 kg and 8 kg squat lifting for three regions of perceived exertions. Rating of Perceived Exertions (RPE).

Body Regions for RPE		Number of Squats					
		4	8	12	16	20	24
		Weights					
		4 kg vs. 8 kg	4 kg vs. 8 kg	4 kg vs. 8 kg	4 kg vs. 8 kg	4 kg vs. 8 kg	4 kg vs. 8 kg
Lower	F	11.73	47.71	54.76	22.65	33.82	3.85
	*p*	0.001	*	*	*	*	0.055
Upper	F	21.87	56.82	40.76	36.96	47.32	87.48
	*p*	*	*	*	*	*	*
Whole body	F	3.27	0.05	18.78	13.36	8.73	0.96
	*p*	0.077	0.821	*	0.001	0.05	0.332

* *p* < 0.01.

**Table 2:** NMPF slopes for the eight muscles of the lower and upper extremity and heart rate. A negative value indicates induced fatigue in muscle. Medial deltoid (MD), anterior deltoid (AD), upper trapezius (UT), supraspinatus (SP), bicep femoris (BF) vastus laterals (VS), gastrocnemius (GS), rectus femoris (RF), and heart rate (HR).

Extremity	Muscle	NMPF Slope	
		4 kg	8 kg
Upper	MD *	−0.44	−0.45
	AD	−0.34	−0.41
	UT	−0.15	−0.12
	SP	−0.15	−0.19
Lower	BF	−0.11	0.23
	VS	−0.19	−0.29
	GS	−0.14	−0.15
	RF *	−0.31	−0.30
Whole body	HR	Heart Rate slope	
		0.7698	0.62

* = Muscle with highest negative slope compared with same extremity.

**Table 3:** Correlation of Borg’s scale ratings with the muscles of the upper and lower extremity and heart rate. Medial deltoid (MD), anterior deltoid (AD), upper trapezius, supraspinatus (SP), bicep femoris (BF) vastus laterals (VS), gastrocnemius (GS), rectus femoris (RF), and heart rate (HR).

Extremity	Muscle Type	Pearson’s *r*	
		4 kg	8 kg
Upper	MD	−0.89	−0.84
	AD	−0.87	−0.88
	UT	−0.95	−0.83
	SP	−0.96 *	−0.87
Lower	BF	−0.84	0.87 *
	VS	−0.95	0.79
	GS	−0.90	−0.83
	RF	−0.78	−0.82
Whole body	HR	0.96	0.95

* = Muscle with highest correlation.

**Table 4:** Three conditions for the central and peripheral rate of perceived exertions.

	Outcome	Implications
1	If the perceived whole body fatigue is higher than upper and lower extremity	Whole body fatigue dominates the peripheral fatigue in squat lifting
2	If upper extremity perceived fatigue is higher than the wholebody and lower extrimty perceived fatigue	Upper body perceived exertion dominates the whole body and lower extremity perceived fatigue in squat lifting
3	If lower body perceived fatigue is higher than the whole body and upper body perceived exertion	Lower body perceived exertion dominates the whole body the and upper body perceived exertion in squat lifting
